# Dynamics of D‐dimer in non‐small cell lung cancer patients receiving radical surgery and its association with postoperative venous thromboembolism

**DOI:** 10.1111/1759-7714.13559

**Published:** 2020-07-13

**Authors:** Lihui Ke, Songping Cui, Shuo Chen, Bin Hu, Hui Li

**Affiliations:** ^1^ Department of Thoracic Surgery, Beijing Chaoyang Hospital Capital Medical University Beijing China

**Keywords:** D‐dimer, non‐small cell lung cancer, surgery, venous thromboembolism

## Abstract

**Background:**

Venous thromboembolism (VTE) occurs at a high rate after lung cancer surgery and can be attributed to various clinical risk factors. Here, we aimed to determine whether early detection of perioperative D‐dimer and risk‐stratified cutoff values would improve the diagnostic efficacy of VTE.

**Methods:**

In this case‐control study, D‐dimer results were acquired from 171 non‐small cell lung cancer (NSCLC) patients preoperatively and at the first, third, and fifth day after surgery. VTE was confirmed by Doppler ultrasonography and computer tomography pulmonary angiography (CTPA). Repeated measures ANOVA was used to analyze how D‐dimer changed with time and the effects of risk factors on D‐dimer levels. We then compared sensitivity, specificity and negative predictive value, using both adjusted and unadjusted cutoff values.

**Results:**

VTE occurred in 23 patients (13.5%) of the study population. D‐dimer levels increased unsustainably after lung cancer surgery (*P* < 0.001) due to a trough on the third day, and patients who had undergone thoracotomy (*P* < 0.001) and those at a more advanced tumor stage (*P* = 0.037) had higher D‐dimer levels. Area under the curve of D‐dimer was greatest on the third day (0.762 [*P* < 0.001, 95% CI: 0.643–0.882]). Applying stratified cutoff values improved the specificity in the video‐assisted thoracoscopy surgery (VATS) (*P* = 0.004) and thoracotomy groups (*P* < 0.001).

**Conclusions:**

D‐dimer levels elevated with fluctuation in NSCLC patients after surgery. Surgical options and tumor stages had an impact on D‐dimer levels. With regard to VTE diagnosis, stratified cutoff values by these two factors showed better accuracy compared with a collective one..

**Key points:**

**Significant findings of the study:**

The changing pattern of perioperative D‐dimer levels in NSCLC patients who received surgical therapy in a major teaching hospital in Beijing, China was revealed.

**What this study adds:**

Risk‐stratified D‐dimer cutoff values adjusted to surgical methods and disease stages would benefit the exclusion of postoperative venous thromboembolism.

## Graphical abstract

D‐dimer increases discontinuously after lung cancer surgery and applying risk‐stratified cutoff values of it makes for better venous thromboembolism (VTE) exclusion.

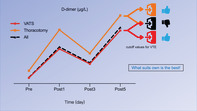

## Introduction

Lung cancer represents as a malignant tumor with high morbidity and mortality worldwide,^1^ which are beyond the world averages, particularly in China.^2^ Prognosis in lung cancer patients is still poor, especially in patients at a more advanced disease stage. Nevertheless, a radical surgery regimen has become the standard therapy for resectable non‐small cell lung cancer (NSCLC) which has contributed to prolonging the lifespan of patients, while operation‐related complications remain risk factors to their prognosis, of which venous thromboembolism (VTE) is non‐negligible.^3^, ^4^, ^5^ Generally, VTE has higher prevalence in cancer patients compared to those with nonmalignant tumors after surgery,^6^, ^7^, ^8^ and, especially, lung cancer ranks among the highest.^5^, ^9^, ^10^, ^11^

Considerable efforts have been made to identify the risk factors related with cancer‐associated VTE, and even though the mechanism behind the occurrence of cancer related VTE has not been fully illustrated,^12^ massive clinical factors that can be summarized as patient‐, treatment‐ and tumor‐related factors jointly act on the development of VTE.^13^ D‐dimer, as an end production of fibrin degradation, is a strong indicator of VTE, which also predicts prognosis and long‐term survival after antitumor therapy.^14^, ^15^, ^16^, ^17^ Many prediction models that integrated D‐dimer as a necessary index have been developed to predict the incidence of cancer‐associated VTE and to stratify patients by different degrees of risk.^18^, ^19^, ^20^, ^21^, ^22^ However, almost every model used preoperative D‐dimer test results as a stationary index, and may therefore have missed the dynamic trend of D‐dimer concentration after surgery which then causes bias when these models are employed. Considering that D‐dimer elevation can be detected in many circumstances such as tumor, surgery, chemoradiotherapy, inflammation, advanced age and so on,^23^ the levels of D‐dimer should be undulatory when cancer patients undergo such antitumor therapy as surgery or chemoradiotherapy.^24^ Evaluating the dynamic change of D‐dimer in combination with clinical risk factors and its association with the incidence of postoperative VTE can help surgeons better hierarchically manage patients and recognize those with a high risk of developing thrombotic events postoperatively. As there are few results on how the D‐dimer concentration changes in patients surgically treated in the thoracic department, in this study, we shed light on the dynamics of D‐dimer in NSCLC patients receiving radical surgical treatment and investigate the relationship between D‐dimer and VTE within certain groups of patients.

## Methods

### Study population

This retrospective study reviewed a total of 265 patients newly diagnosed with NSCLC according to pathological results from July 2016 to December 2017, admitted to Beijing Chaoyang Hospital for primary regimen of surgical treatment. A total of 94 patients were excluded for (i) being diagnosed with VTE within three months before surgery; (ii) recent oral administration or subcutaneous injection of anticoagulant agencies; and (iii) incomplete demographic data. A total of 171 patients were enrolled into the study for analysis. None of the included patients received neoadjuvant chemotherapy before surgery. The clinical characteristics and D‐dimer test results required for analysis were obtained from electronic medical records, including age, gender, body mass index, surgical method, surgery procedure, duration of operation, intraoperative blood loss, pathological type, TNM stage and VTE event. A VTE event included deep venous thrombosis (DVT) and pulmonary embolism (PE). Doppler ultrasonography of the lower extremity vein was executed to detect DVT in all patients pre‐ and postoperatively. Computer tomography pulmonary angiography (CTPA) was performed to confirm PE in patients who manifested clinical symptoms or were deemed to be high risk on surgical evaluation. Blood samples were taken at four points of time: the day before surgery, the first, third, and fifth day after surgery. All samples were delivered for D‐dimer assay by immunoturbidimetry (INNOVANCE D‐dimer, SIEMENS) within two hours after collection from veins in the forearm. Surgical options (video‐assisted thoracoscopic surgery [VATS] or open surgery) were determined on the basis of tumor size and location, as well as patients' comorbidities and endurance against relevant surgical procedures. If a difficult situation arose in the process of VATS and traditional thoracotomy was the treatment of choice, then it was manually documented as open surgery in those patients' medical records. All data was collected up to the time of discharge from hospital. The consents were waivered for the retrospective nature of this study. This retrospective study was approved by the Beijing Chao‐yang Hospital Institutional Review Board (ID: 2017‐Ke‐1).

### Statistical analysis

The baseline characteristics were expressed as mean ± standard derivation (SD), ratio or median with interquartile range (IQR) accordingly. Multiple imputation was applied when perioperative D‐dimer results were vacant.^25^ The D‐dimer concentration was Ln‐transformed before statistical analysis due to the skewed distribution. Repeated measures ANOVA was performed, and F statistics were used to detect the time effects on perioperative D‐dimer levels. A post hoc test was performed to analyze discrepancy of dynamics between different outcome subgroups and differences ascribed to surgical options, stage and VTE within each point of time, where the *P*‐value was adjusted by the Bonferroni method. The time effect on D‐dimer levels were described as changes compared with baseline D‐dimer before operation, as the parameter was Ln‐transformed beforehand, so the average increments of D‐dimer concentration (Δ) were described as relative. Receiver‐operating‐characteristic (ROC) curve was used to discriminate VTE patients and non‐VTE patients. The optimum cutoff value was approached by Youden's J statistic. We also calculated sensitivity, specificity and negative predictive value (NPV) according to optimum cutoff values and then used Chi‐square test and Fisher's exact test to compare these parameters between groups. Results having a two‐tailed *P*‐value <0.05 were considered to be statistically significant. All statistical analyses were conducted using IBM SPSS statistics version 22.0 (Armonk, NY: IBM Corp).

## Results

### Patient characteristics

A total of 171 patients were retrospectively analyzed in this study. Each patient had at least three D‐dimer detections out of four time‐points. All 637 blood samples were drawn for analysis (47 missed in total). The median age at diagnosis was 58 years (range: 35–82 years). The number of male patients was 75 (43.9%) of the whole population. All patients received radical surgical therapy with complete resection. Thoracotomy was performed in a total of 25 (14.6%) patients, one of whom was moved to thoracotomy after the original VATS procedure failed. Most patients had a single pulmonary lobe removed and only two patients underwent pneumonectomy. In terms of histological type, 142 (83.0%) of the tumors were adenocarcinomas and the others (*n* = 29, 17%) were squamous cell carcinomas. For pathological staging, there were 127 (74.3%) patients with early stage (0 + I) disease, and 44 (25.7%) with advanced stage (II + III) disease. Until discharge, 23 (13.5%) patients were diagnosed with VTE, including 22 (12.9%) with DVT alone and one (0.6%) with both DVT and PE (Table 1).

**Table 1:** Clinical characteristics and D‐dimer levels of the NSCLC patients (*n* = 171)

Characteristics	
Age at diagnosis (years)^†^	58 (35–82)
Gender (male)	75 (43.9)
BMI (kg/m^2^)^‡^	23.8 ± 3.2
Surgical approach	
Thoracotomy	25 (14.6)
VATS	146 (85.4)
Surgery procedure	
Sublobar resection	21 (12.3)
Lobectomy	148 (86.5)
Pneumonectomy	2 (1.2)
Duration of operation (minutes)^‡^	170.0 ± 47.3
Intraoperative blood loss (mL)^¶^	100 (100–200)
Histological type	
Adenocarcinoma	142 (83.0)
Squamous cell carcinoma	29 (17.0)
TNM stage	
0	40 (23.4)
I	87 (50.9)
II	24 (14.0)
III	20 (11.7)
VTE event	47 (10.8)
DVT alone	22 (12.9)
PE alone	0 (0)
PE + DVT	1 (0.6)
D‐dimer concentration (μg/L)^§^	
Pre‐D‐dimer	200 (130–380)
Post 1‐D‐dimer	1200 (750–1890)
Post 3‐D‐dimer	680 (470–1100)
Post 5‐D‐dimer venous	1840 (1230–2660)

BMI, body mass index; DVT, deep vein thrombosis; NSCLC, non‐small cell lung cancer; PE, pulmonary embolism; VATS, video‐assisted thoracoscopic surgery; VTE, venous thromboembolism.

^†^ Median (range).

^‡^ Mean ± SD.

^¶^ Mean (interquartile range).

^§^ Serum D‐dimer concentration was tested prior to surgery and on the first, third, and fifth day postoperatively and is expressed as mean (interquartile range). Other characteristics without special note are expressed as *n* (%).

### Perioperative D‐dimer changes according to clinical factors

**Figure 1:**
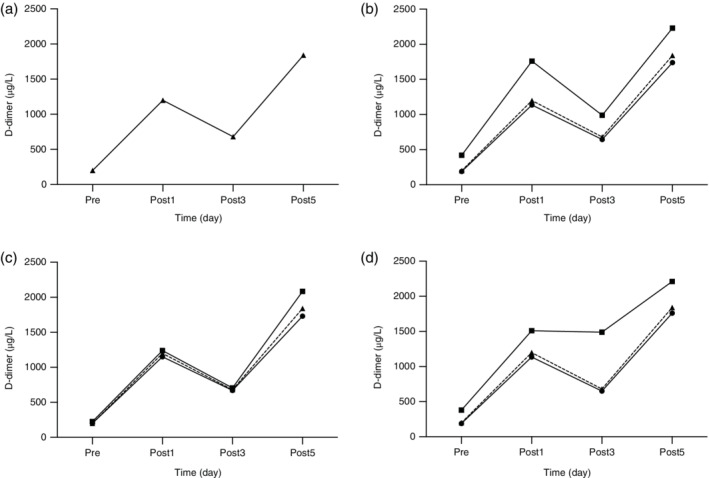
(**a**) Hemostatic profile indicated by perioperative D‐dimer concentration (μg/L) of whole study population; and (**b**) patients divided into different subgroups including surgical approaches. 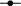, VATS; 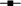, Thoracotomy; 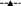, All. (**c**) Tumor stages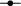, 0 + I; 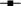, II + III; 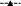, All (**d**) Venous thromboembolism 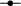, nVTE; 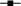, VTE; 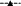, All. The change of D‐dimer in the whole population is shown as a dotted line in the other three figures, respectively.

Overall, the D‐dimer level increased significantly after surgery (*P* < 0.001), but not constantly as it met a slight slump on third day, even though it was still higher than that before surgery (Δ = 1.12, 95% CI: 0.97–1,27, *P* < 0.001) (Fig 1a). D‐dimer level reached its highest on the fifth day after surgery (Δ = 2.05, 95% CI: 1.92–2.18, *P* < 0.001). When it came to comparison between groups (Table 2), D‐dimer levels were significantly higher in the thoracotomy group (*n* = 25) than the VATS group (*n* = 146) (*P* < 0.001), and 0 + I stage group (*n* = 127) had higher levels than those in the II + III stage group (*n* = 44) (*P* = 0.037). To be significant, the age distribution between each pair of subgroups was well balanced (see Supplement 1), and there were no significant differences contributed to other clinical factors. Intriguingly, by different grouping, there were the same tendencies of D‐dimer levels as in respective subgroups as in the whole study population (Fig 1b and c). At each timepoint, patients receiving thoracotomy had a much higher D‐dimer concentration postoperatively. As for stage grouping, there was a significant elevation of D‐dimer levels in II + III groups only prior to surgery (*P* = 0.006), while no significant impact was seen on postoperative D‐dimer levels (Table 3).

**Table 2:** Comparisons of D‐dimer levels by different grouping during the whole study

Factors	Cases (*n*)	F	*P*‐value
Age		0.774	0.380
≤58	87		
>58	84		
Gender		3.489	0.064
Male	75		
Female	96		
Surgical approach		20.170	<0.001
Thoracotomy	25		
VATS	146		
Surgery procedure		0.846	0.431
Sublobar resection	21		
Lobectomy	148		
Pneumonectomy	2		
Duration of operation		1.712	0.193
≤170 minutes	90		
>170 minutes	81		
Intraoperative blood loss		0.761	0.384
≤100 mL	98		
>100 mL	73		
Histological type		0.190	0.663
Adenocarcinoma	142		
Squamous cell carcinoma	29		
TNM stage		4.424	0.037
0 + I	127		
II + III	44		
VTE event		13.879	<0.001
Yes	23		
No	148		

Only factors between which D‐dimer levels changed significantly differently with time were included for further pairwise comparison.

VATS, video‐assisted thoracoscopic surgery; VTE, venous thromboembolism.

**Table 3:** The differences of D‐dimer levels (μg/L) at four points of time according to various factors

	VATS	Thoracotomy	*P*‐value	0 + I	II + III	*P*‐value	Non‐VTE	VTE	*P*‐value
Pre	190 (130–338)	420 (200–735)	<0.001	200 (128–340)	255 (170–478)	0.006	190 (130–340)	380 (200–500)	0.023
Post1	1135 (728–1715)	1760 (1085–3100)	0.003	1150 (720–1890)	1240 (803–1943)	0.415	1135 (730–1760)	1510 (1090–3700)	0.005
Post3	645 (428–978)	990 (800–1665)	<0.001	670 (410–1080)	705 (573–1130)	0.170	650 (443–988)	1490 (840–1970)	<0.001
Post5	1740 (1225–2518)	2230 (1492–4193)	0.002	1730 (1210–2570)	2084 (1339–3473)	0.097	1760 (1215–2563)	2212 (1146–3567)	0.071

VATS, video‐assisted thoracoscopic surgery; VTE, venous thromboembolism.

### D‐dimer variation according to VTE outcomes

In 23 patients (13.5%) diagnosed with VTE after surgery, there were significantly higher levels of D‐dimer in comparison to patients without VTE (*P* < 0.001), but at the fifth day no significant differences were discovered (*P* = 0.071). These two subgroups shared a similar growth trend in D‐dimer levels with the whole population. However, in the VTE group (Table 4), there was a relatively steadier variation of D‐dimer postoperatively (Fig 1d) because no differences were found between the first and third day (*P* = 0.316), and between the first and fifth day (*P* = 0.665).

**Table 4:** Changes of D‐dimer between different timepoints in the VTE group and n‐VTE group

	VTE group	n‐VTE group
*P*‐value	(*n* = 23)	(*n* = 148)
Pre‐: Post 1	<0.001	<0.001
Pre‐: Post 3	<0.001	<0.001
Pre‐: Post 5	<0.001	<0.001
Post 1: Post 3	0.319	<0.001
Post 1: Post 5	0.665	<0.001
Post 3: Post 5	<0.001	<0.001

### ROC analysis on relationship between D‐dimer and VTE

When taking the whole study population into consideration, the area under the curve (AUC) was greatest, which was 0.762 (*P* < 0.001, 95% CI: 0.643–0.882) on the third day after lung resection, and the cutoff value of D‐dimer was 835 μg/L (sensitivity = 78.3%, specificity = 68.2%). The AUC was then calculated separately in different subgroups divided by surgical method and staging. The AUC was higher in the VATS group (AUC = 0.727 [*P* = 0.007, 95% CI: 0.561–0.894]) than in the thoracotomy group (AUC = 0.676 [*P* = 0.153, 95% CI: 0.435–0.918]), but with no significant difference (Z = 0.339, *P* = 0.051). The AUC for 0 + I stage group was 0.784 (*P* < 0.001, 95% CI: 0.634–0.934) and 0.705 (*P* = 0.057, 95% CI: 0.494–0.916) for II + III stage group, and there was still no significant difference between the two groups (Z = 0.599, *P* = 0.079). The differences of sensitivity and specificity were not significant between subgroups when using respective cutoff values, as well as when it came to comparison between subgroups using a fixed cutoff value derived from the whole population (Table 5). Using respective cutoff values in each subgroup did not significantly improve the diagnostic accuracy except for specificity in the VATS group (*P* = 0.004) and the thoracotomy group (*P* < 0.001). In terms of NPV, NPV of patients at early disease stage was 97.3% and 96.4% by two kinds of cutoff values (*P* = 1.000), and in patients with stage II + III, NPV was 91.7% and 91.3%, respectively (*P* = 1.000). Patients receiving thoracotomy had the lowest NPV (75.0%; 66.7%) compared with those undergoing VATS (91.7%; 90.0%).

**Table 5:** Accuracy of D‐dimer for VTE resulting from different cutoff values

Subgroups	Parameter	Cutoff^†^	Cutoff2^‡^	*P*‐value
VATS	Sensitivity	5/15 (33.3%)	5/15 (33.3%)	1.000
	Specificity	110/131 (84.0%)	90/131 (68.7%)	0.004
	NPV^§^	110/120 (91.7%)	90/100 (90.0%)	0.669
Thoracotomy	Sensitivity	3/8 (37.5%)	6/8 (75.0%)	0.313
	Specificity	15/17 (88.2%)	4/17 (23.5%)	<0.001
	NPV	15/20 (75.0%)	4/6 (66.7%)	1.000
0 + I	Sensitivity	12/14 (85.7%)	11/14 (78.6%)	1.000
	Specificity	73/113 (64.6%)	80/113 (70.8%)	0.319
	NPV	73/75 (97.3%)	80/83 (96.4%)	1.000
II + III	Sensitivity	7/9 (77.8%)	7/9 (77.8%)	1.000
	Specificity	22/35 (62.9%)	21/35 (60.0%)	0.806
	NPV	22/24 (91.7%)	21/23 (91.3%)	1.000

^†^ Each subgroup used the cutoff value from ROC analysis that was performed respectively (VATS: 1250 μg/L; thoracotomy: 1745 μg/L; 0 + I: 755 μg/L; II + III: 870 μg/L).

^‡^ Each subgroup used the same cutoff value ROC analysis from the whole population (835 μg/L).

^§^ NPV, negative predictive value.

## Discussion

In this retrospective study, we aimed to detect the early changes of D‐dimer levels in NSCLC patients after lung resection and explore the diagnostic capability of D‐dimer for VTE. We also investigated the influences of different clinical factors on D‐dimer dynamics and whether divergent effects existed on diagnostic efficacy between different subgroups by these factors. Overall, from 637 blood samples in 171 patients, we observed a nonlinear increase in serum D‐dimer during the first five days postoperatively, and patients receiving thoracotomy procedures, or with advanced disease, had higher levels. Despite the differences in coagulation state, the probability of patients developing VTE showed no significant differences in different clinical contexts. However, using stratified cutoff values of D‐dimer would improve the accuracy of VTE events, especially in exclusive ability.

Cancer‐associated VTE can be attributed to heterogeneous risk factors, including tumor‐, patient‐ and treatment‐related factors. A large number of lung cancer patients are admitted to hospital at an advanced stage of disease, who were in poor physical health when their cancer was confirmed, and this results in some of them not being suitable for curative surgeries and they have had to accept palliative chemoradiotherapy. However, early screenings have allowed more curative cases with the help of an active surgical strategy;^26^, ^27^ in addition, adjuvant therapy also helps to prolong the life span of lung cancer patients, especially those at an advanced stage of disease at diagnosis.^28^ Until now, more efforts have been made to investigate antitumor treatment associated VTE in nonsurgical patients.^9^, ^10^, ^29^ It is therefore necessary to focus on postoperative VTE that encompasses a patient's tumor burden and operational stress, which may be heterogeneous from other situations where chemical therapy has played a more robust role.

To identify those at high risk of developing VTE, a consistent monitoring of positive symptoms and blood markers in alliance with clinically relevant risks is needed,^30^ and because many clinical cases are asymptomatic, an effective surveillance of coagulation indicators is more important. The hematological state alters a lot after surgery, which can be indicated by various blood biomarkers including coagulation factor (F) VIII, fibrinogen and soluble P‐selectin (sP‐selectin).^31^ Elevated FVIII may be of predictable value for the recurrence of VTE, but its predictive role has not been consistent among different results and may be not simply attributed to short‐term reaction because of its persistent elevation over time.^32^ D‐dimer is considered as a most powerful indicator of venous thromboembolism and arterial thrombotic diseases. Although for what length of time high levels of D‐dimer remain varies among studies,^33^, ^34^, ^35^ mainly caused by inconsistent follow‐up period in different researches, we hypothesized that for surgical patients, the high levels of D‐dimer will reduce to baseline status within a month, which is shorter than those additionally receiving subsequent adjuvant chemotherapy.^24^ D‐dimer levels were higher than baseline in patients who developed VTE after surgery, and studies have shown that preoperative levels of D‐dimer had an impact on survival of NSCLC patients.^16^ However, it seems irresponsible to omit the fluctuations of D‐dimer levels after surgery. Actually, increased attention is being paid to long‐term D‐dimer level changes and the relationship with VTE,^36^ and in our experience, a more meticulous tracking of D‐dimer levels can depict a more detailed hematological profile, and can be achieved by following the level changes closely in adjacent days instead of at subsequent relatively longer intervals. Given the purpose of cutting healthcare costs and future clinical practicability, we measured D‐dimer concentration before surgery and the first, third, and fifth day after surgery. We detected increasing levels of D‐dimer with an unexpected changing pattern during the first five days after lung resection in this study. But whether this kind of tracking strategy would better reveal the procoagulant variation, especially during the days immediately following lung cancer surgery, needs to be further investigated.

In this study, two major factors affecting postoperative D‐dimer, namely advanced disease stage and the traditional surgical option, were identified. It is acknowledged that more advanced tumor stage is an independent contributor to VTE development.^37^ As for patients undergoing thoracotomy, they usually experience a longer surgical procedure by a more invasive method, which can lead to more intraoperative blood loss and longer stasis in pulmonary vessels than patients undergoing thoracoscopic surgery, which is accompanied by a higher possibility of postoperative complications such as persistent air leak, atelectasis and a prolonged period of bed rest.^38^ As a result, they tend to be in a more procoagulant state and have higher D‐dimer levels, leaving their counterparts who are treated by less invasive methods with lower levels of D‐dimer, and which were even equivalent with the whole study population (Fig 1b) in this study. Actually, with the help of early screenings, the proportion of patients who are now surgically treated by open surgery and pneumonectomy is reducing, but still, more focus should be placed on them to prevent missing those at higher risk.

It is always difficult for clinicians to identify high‐risk patients by comprehensive analysis of various factors. When employing D‐dimer, using a stationary cutoff value for unselected patients will cause huge bias as it is influenced by various confounders, especially tumor stage and surgical method for patients in this study. Taking age as an example, a cutoff value adjusted to age substantially increases the utility of D‐dimer to exclude pulmonary embolism, which is quite clinically relevant because this strategy presents optimal performance in elder patients when they are at low risk.^39^ However, in our study, the age distribution was well balanced between interesting subgroups. Therefore, we performed a separate ROC analysis based on different risk factors in order to determine the divergent cutoff values in each set of groups. Not surprisingly, there was an elevated diagnostic threshold of D‐dimer in patients undergoing more invasive procedures, or who were at a more advanced stage of disease, but no significant differences in sensitivity and NPV were seen between subgroups (Table 5). On the other hand, soley using the cutoff value that was based on the whole population slightly reduced the diagnostic efficacy, especially with regard to exclusion of a negative outcome. These results prove the necessity of predicting whether patients were to have a VTE event on the basis of various clinical circumstances in order to bring about at least diagnostic ability and better exclusive accuracy.

In clinical practice, patients where there is a high suspicion of VTE based on D‐dimer results, or by their relevant symptoms, require further examination. DVT is mainly confirmed by ultrasound and PE by CTPA or pulmonary angiography, which can bring associated high medical care expenditure and procedure‐related life threat to patients. In particular, for patients who have received lung resection, respiratory functions are impaired and hemodynamics are compromised following surgery, both of which multiply the mortality during injection of a contrast agent.^40^ Because such postoperative complications occur more frequently in thoracotomy patients, it is more important to be selective in carrying out examinations for diagnosis or exclusion, which represents as an ever‐popular goal that individual testing strategy can provide a better solution to achieve. D‐dimer is usually used for excluding a diagnosis of thromboembolic disease, and is a simple strategy which can be applied whenever required, thus avoiding unnecessary intervention if patients are at low risk of VTE, and more importantly, identifying those who will benefit most from prothrombotic treatment. Ingrid *et al*.^20^ proposed a clinical prediction model for cancer‐associated VTE incorporating only two parameters: tumor type and D‐dimer concentration, which aimed to discriminate between patients who have a different propensity to develop VTE in mixed cancer cohorts, but it seems underpowered when only lung carcinoma is taken into consideration. Khorana, CATS, and PROTECHT risk models which use more clinical factors and biomarkers also demonstrate an unsatisfactory predictive efficacy within the cancer specific category.^41^ So, developing models that can be more specific in single category of malignancy for VTE prediction is essential. In thoracic surgery patients, a prospective study demonstrated that the Caprini risk assessment model (RAM) can be applied safely,^42^ but it contains no laboratory parameters.

Additionally, prediction models also have limitations in evaluating the possibility of VTE during a relatively short time span after major surgery, even if an attempt is made to incorporate dynamic parameters to create a so‐called real‐time predictive model. Consider that the procoagulant factors are mostly determined (in particular like the surgical method for surgery patients) soon after initiation of antitumor treatment for individual patients, so in these cases, the significance of these models is undermined. The Caprini RAM is mainly used for risk stratification and indicating the launch of anticoagulant treatment in a surgical context, and it has been demonstrated to reduce symptomatic VTE by extended prophylaxis within two months after discharge from the thoracic department.^43^ However, in most cases, scores alter only as patients finish their surgical procedure, which means that it is mainly determined by surgical procedure, but not VTE development, in most in‐patient cases. Based on this acknowledgement, we preferred to tracing the alterable parameter, that is D‐dimer, as a major indicator to complement imaging examinations. In this study, the level of D‐dimer on the third day after surgery was demonstrated to have the strongest diagnostic efficacy, which appears to lack proper explanation in biological rationale. It was not until the imaging diagnosis was confirmed that most patients were administered pharmacological thromboprophylaxis in this study, and therefore we were unable to directly compare whether this monitoring strategy outweighed the implementation of Caprini RAM. However, it served as a reminder that for lung cancer patients who received curative resection, early intervention for VTE may be beneficial, which needs to be verified in a prospective and randomized study.

Some drawbacks of this study, in particular the small population size and retrospective nature, impeded some conclusive interpretations. The number between subgroups was not equal, which is likely to cause bias. Also, we were unable to develop a risk model because of multicollinearity of the consecutive data and lack of follow‐up. There have been several studies that attempted to identify independent risk factors of VTE in patients who received thoracic surgery for treating lung cancer, but the D‐dimer concentration was enrolled into multivariate analysis with other variables.^8^, ^13^ In this study, we carried out a separate ROC analysis by factors that affected D‐dimer levels to investigate different impacts of clinical factors on VTE development by stratified application of D‐dimer cutoff values, which elevated the diagnostic capability of the D‐dimer testing results during the first five days after surgery. However, only the D‐dimer on the third day after surgery achieved a satisfying diagnostic value. Whether this kind of consecutive measurement of D‐dimer and hierarchical classification of cutoff values could best identify a high‐risk population immediately after lung cancer surgery, and instruct the initiation of prophylactic anticoagulation, has yet to be further investigated. Indeed, to extend the following timeline, other dynamic risk factors may reasonably impact on D‐dimer concentration and modify VTE risk.^44^ Ongoing prospective research aims to assess the risk profile by adopting a wider range of biomarkers and track their changes, in combination with other clinical risk factors in order to create a risk stratification tool employing simple biomarkers to provide an appropriate thromboprophylaxis strategy for NSCLC patients after commencing antitumor treatment.^45^ Actually, incorporating follow‐up research would include more patients who received adjuvant therapy with more complete tracking of VTE development in the full process of treatment, which is a further step for us. Although our study enrolled a limited number of patients and only assessed VTE event until discharge, we provided a similar and more simple way to carry out dynamic and stratified evaluation of VTE risk and proved its availability and efficacy for thoracic surgery patients.

In conclusion, this study depicted a procoagulant profile by D‐dimer results in NSCLC patients in their early recovery from surgical intervention. The dynamics of D‐dimer in VTE patients represented a differential trend from others, but there were no significant differences of trend between patients of different tumor stage and different surgical options, except for quantitative imparity. However, using stratified rather than collective cutoff values is of benefit for the exclusion and diagnosis of VTE events in lung cancer patients in the early days after curative resection.

## Disclosure

All authors declare that there are no conflicts of interest.

## Supporting information


**Appendix** **S1:** Supporting Information
