# Supplementary material for: Dynamics of D‐dimer in non‐small cell lung cancer patients receiving radical surgery and its association with postoperative venous thromboembolism
Source: Thorac Cancer. 2020 Jul 13;11(9):2483–92. doi: 10.1111/1759-7714.13559 (PMC7471045; doi:10.1111/1759-7714.13559)
Supplement: Supplementary file 1 — Appendix S1: Supporting Information [file TCA-11-2483-s001.docx]

**Supplement 1** Distribution of age, operating time and blood loss between different risk factors

|  | VATS | Thoracotomy | P | 0+I | Ⅱ+III | P |
| --- | --- | --- | --- | --- | --- | --- |
| Age (years) | 58.4±8.4 | 60.1±11.0 | 0.388 | 59.0±8.6 | 57.6±9.5 | 0.366 |
| Duration of operation (mins) | 165.0±41.8 | 191.8±68.4 | 0.009 | 163.7±42.2 | 183.6±57.7 | 0.016 |
| Intraoperative blood loss (ml) | 100 (100-200) | 300 (100-600) | <0.001 | 100 (100-200) | 175 (100-300) | 0.001 |

VATS, video-assisted thoracoscopic surgery
